# Stability indicating HPLC method for the simultaneous determination of moxifloxacin and prednisolone in pharmaceutical formulations

**DOI:** 10.1186/1752-153X-6-94

**Published:** 2012-09-04

**Authors:** Syed Naeem Razzaq, Islam Ullah Khan, Irfana Mariam, Syed Saleem Razzaq

**Affiliations:** 1 Department of Chemistry, Government College University, Lahore, 54000, Pakistan; 2 Department of Chemistry, Queen Marry College, Lahore, 54000, Pakistan; 3 Medipharm Pharmaceuticals Kot Lakhpat, Lahore, 54000, Pakistan

**Keywords:** Reverse phase liquid chromatography, Moxifloxacin, Prednisolone, Degradation products, ICH guidelines etc

## Abstract

**Background:**

A simple, specific, and fast stability indicating reverse phase liquid chromatographic method was established for instantaneous determination of moxifloxacin and prednisolone in bulk drugs and pharmaceutical formulations.

**Results:**

Optimum chromatographic separations among the moxifloxacin, prednisolone and stress-induced degradation products were achieved within 10 minutes by use of BDS Hypersil C8 column (250 X 4.6 mm, 5 μm) as stationary phase with mobile phase consisted of a mixture of phosphate buffer (18 mM) containing 0.1% (v/v) triethylamine, at pH 2.8 (adjusted with dilute phosphoric acid) and methanol (38:62 v/v) at a flow rate of 1.5 mL min^-1^. Detection was performed at 254 nm using diode array detector. The method was validated in accordance with ICH guidelines. Response was a linear function of concentrations over the range of 20–80 μg mL^-1^ for moxifloxacin (r2 ≥ 0.998) and 40–160 μg mL^-1^ for prednisolone (r2 ≥ 0.998). The method was resulted in good separation of both the analytes and degradation products with acceptable tailing and resolution. The peak purity index for both the analytes after all types of stress conditions was ≥ 0.9999 indicated a complete separation of both the analyte peaks from degradation products. The method can therefore, be regarded as stabilityindicating.

**Conclusions:**

The developed method can be applied successfully for simultaneous determination of moxifloxacin and prednisolone in pharmaceutical formulations and their stability studies.

## Introduction

**Figure 1:**
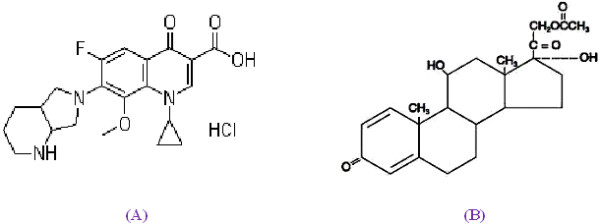
Chemical Structures of Moxifloxacin HCl (A) and Prednisolone Acetate (B).

Moxifloxacin hydrochloride is chemically designated as 1-Cyclopropyl-6-fluoro-1,4-dihydro-8-methoxy-7-[(4aS,7aS)-octahydro-6 H-pyrrolo[3,4-b]pyridin-6-yl]-4-oxo-3-quinolinecarboxylic acid hydrochloride [Figure 1. It is a broad-spectrum antibiotic that functions by inhibiting DNA gyrase, a type II topoisomerase, and topoisomerase IV enzymes [1] necessary to separate bacterial DNA, thereby inhibiting cell replication. It is used for bacterial conjunctivitis, keratitis, pre & post operative conditions to control the infections of the eyes. Prednisolone acetate [Figure 1 chemically designated as 11β 17, 21-trihydroxypregna-1,4-diene-3,20-dione 21-acetate is a corticosteroid, used principally for steroid-responsive inflammatory ocular conditions for which a corticosteroid is indicated and where bacterial ocular infection or a risk of infection exists [2]. Both moxifloxacin and prednisolone have been analysed by various techniques either alone or in combination with other drugs. The analytical methods existed for moxifloxacin hydrochloride included determination by spectrophotometry [3-5], and high performance liquid chromatography [6,7]. The analytical methods existed for prednisolone included determination by micellar electrokinetic chromatography [8], spectrophotometry [9], high performance liquid chromatography [10,11], and thin layer chromatography densitometry [12].

The combination of moxifloxacin and prednisolone has not been adopted by any official pharmacopoeia (USP, BP or EP etc.). An extensive review of the literature did not revealed any stability indicating HPLC method for simultaneous determination of both drugs. Therefore, attempts were made to develop and validate simple, precise, and sensitive, isocratic reverse phase stability indicating high performance liquid chromatographic method for simultaneous determination of both drugs and their degradation products in pharmaceutical formulations. We are currently engaged in binary combination analysis of different classes of drugs in pharmaceutical formulations and in human plasma [13-23].

## Experimental

### Chemicals and reagents

Reference standards of moxifloxacin hydrochloride and Prednisolone acetate with stated purity of 99.97 and 99.46% respectively were obtained from Schazoo Zaka Laboratories (Lahore, Pakistan). Occumox P and Moftrex P eye drops (claimed to contain 5 mg per mL of moxifloxacin and 10 mg per mL of prednisolone) were used in this study. Occumox P eye drops contains 0.02% w/v benzalkonium chloride as preservative in sterile aqueous base and Moftrex P eye drops contains 0.25% w/v hydroxypropylmethylcellulose (HPMC) as preservative in the sterile aqueous base. Methanol (HPLC grade), potassium dihydrogen phosphate, phosphoric acid, triethylamine, sodium hydroxide, hydrochloric acid and hydrogen peroxide (analytical reagent grade) were from M.S Traders Lahore, Pakistan (Fluka origin). Double distilled water was used throughout the analysis. Mobile phase was filtered using 0.45μm nylon filters by Millipore (USA).

### Equipment and chromatographic conditions

The HPLC system consisted of Shimadzu LC-20A system (Kyoto, Japan) equipped with model LC-20AT pump, SPD-M20A Diode array detector (set at 254 nm), and DGU-20A5 online degasser, and a Rheodyne injection valve with a 20 μL loop. Peak areas were integrated using a Shimadzu LC solution (version 1.227) software program. The experimental conditions were optimized on a BDS Hypersil C8 column (250 X 4.6 mm, 5 μm) at room temperature. Mobile phase consisted of methanol and 18 mM phosphate buffer (pH 2.8) in the ratio of (62:38 v/v, respectively). The phosphate buffer was prepared by taking 2.448 g of potassium dihydrogen phosphate in 1000 mL of water. Triethylamine (1 mL) was added to it and pH was then adjusted to 2.8 using phosphoric acid. Flow rate of the mobile phase was 1.5 mL min^-1^ and all chromatographic experiments were performed at room temperature (25°C ± 2°C).

### Preparation of standard stock solution

Stock solution was prepared to reduce the number of repetitive operations involved and hence, the chances of human or experimental error. Moreover direct weighing of moxifloxacin (50 μg mL^-1^) and prednisolone (100 μg mL^-1^) to prepare the working standard solution cannot be performed with sufficient accuracy. Standard stock solution of moxifloxacin (1250 μg mL^-1^) and prednisolone (2500 μg mL^-1^) was prepared by accurately weighing 31.25 mg moxifloxacin and 62.5 mg prednisolone dissolved in 5–7 mL methanol in 25 mL volumetric flask and then up to the mark with mobile phase. The stock solution was used to prepare the working standard solution of moxifloxacin and prednisolone. Stock solution of moxifloxacin and prednisolone was also used to prepare working solutions for accuracy, precision, linearity (0.4 mL-1.6 mL of the stock solution diluted to 25 mL with mobile phase), forced degradation studies (1 mL of stock solution + 1 mL of acid or base solution diluted to 25 mL with mobile phase) and robustness etc.

### Preparation of standard solution

1 mL of the standard stock solution was diluted to 25 mL with mobile phase to prepare working standard solution having concentration equal to 50 μg mL^-1^ of moxifloxacin and 100 μg mL^-1^ of prednisolone. The solution was filtered through 0.45μm nylon filter before analysis.

### Preparation of sample solution

1 mL commercial eye drops (composition 5 mg mL^-1^ moxifloxacin and 10 mg mL^-1^ prednisolone) were diluted to 100 mL with mobile phase to obtain concentration equal to 50 μg mL^-1^ of moxifloxacin and 100 μg mL^-1^ of prednisolone. The solution was filtered through 0.45 μm nylon filter before analysis.

### Linearity

Linear calibration plots of the proposed method were obtained over concentration ranges of 20–80 μg mL^-1^ (20, 30, 40, 50, 60, 70 and 80 μg mL^-1^) for moxifloxacin and 40–160 μg mL^-1^ prednisolone (40, 60, 80, 100, 120, 140 and 160 μg mL^-1^). Each solution was prepared in triplicate.

### Accuracy

Accuracy of an analytical procedure is the closeness of agreement between accepted conventional true values (reference values) and the values found. Accuracy of the developed method was determined by two ways i) standard addition method and ii) analysis of synthetic mixtures of moxifloxacin and prednisolone eye drops. In the standard addition method known quantities (50, 100 and 150%) of moxifloxacin and prednisolone were supplemented to the sample solution previously analysed and then experimental and true values were compared. In the synthetic mixture method known quantities (50, 100 and 150%) of moxifloxacin and prednisolone of known purity have been spiked to the placebo components (benzalkonium chloride and sodium chloride in aqueous base). Synthetic mixture (100% nominal analytical concentration) of moxifloxacin (50 μg mL^-1^) and prednisolone (100 μg mL^-1^) was prepared by mixing moxifloxacin (500 mg), prednisolone (1000 mg), benzalkonium chloride (0.02 g) as preservative and sodium chloride (0.3 g) in 100 ml of purified water for 30 minutes using magnetic stirrer. Three levels of synthetic mixtures were prepared corresponding to 50, 100 and 150% of nominal analytical concentration (50 μg mL^-1^ of moxifloxacin and 100 μg mL^-1^ of prednisolone) and analysed by the developed method.

### Precision

Repeatability was studied by determination of intra-day and inter-day precision. Intra-day precision was determined by injecting five standard solutions of three different concentrations on the same day and inter-day precision was determined by injecting the same solutions for three consecutive days. Relative standard deviation (RSD%) of the peak area was then calculated to represent precision.

### Specificity (stress testing)

Stress testing was carried out using different ICH prescribed stress conditions such as acidic, basic, oxidative, thermal and photolytic stresses. All stress studies were performed in 25 mL volumetric flask.

#### Acid degradation studies

Acid degradation study was performed in versatile environmental test chamber (Sanyo, Japan) at 40°C/75% RH using 5 M HCl. For this purpose, 1 mL of the standard stock solution was taken in 25 mL volumetric flask. One milliliter of 5 M HCl was added in the flask and kept in versatile environmental test chamber at 40°C/75% RH for 16 h. After completion of the stress the solution was neutralized by using 5 M NaOH and completed up to the mark with mobile phase.

#### Base degradation studies

Base degradation study was performed at 22°C/58% RH using 5 M NaOH. For this purpose, 1 mL of the standard stock solution was taken in 25 mL volumetric flask. One milliliter of 5 M NaOH was added in the flask and kept at 22°C/58% RH for forty minutes. After completion of the stress the solution was neutralized by using 5 M HCl and completed up to the mark with mobile phase.

#### Oxidative degradation studies

Oxidative degradation study was performed in versatile environmental test chamber (Sanyo, Japan) at 40°C/75% RH using 6% H_2_O_2_. For this purpose, 1 mL of the standard stock solution was taken in 25 mL volumetric flask. One milliliter of 6% H_2_O_2_ was added in the flask and kept in versatile environmental test chamber at 40°C/75% RH for 16 h. After completion of the stress, the 25 mL flask was completed up to the mark with mobile phase.

#### Thermal degradation studies

Thermal degradation studies were performed at two different temperatures i) in versatile environmental test chamber (Sanyo, Japan) at 40°C/75% RH and ii) in oven (Gallenkamp, UK) at 105°C (dry heat thermolysis). For this purpose, 1 mL of the standard stock solution was taken in two different 25 mL volumetric flasks and kept in versatile environmental test chamber at 40°C/75% RH for 144 h and 288 h. After specified time, the 25 mL flasks were completed up to the mark with mobile phase. For dry heat thermolysis, 62.5 mg moxifloxacin and 125 mg of prednisolone were mixed for 30 minutes in glass petty dish with spatula and placed in oven at 105°C for seven hours. After specified time, 5–7 mL methanol was added to the powder mixture and up to mark 50 mL with mobile phase. 1 mL of this solution was further diluted to 25 mL with mobile phase.

#### Photolytic degradation studies

For photolytic degradation study 1 mL of the standard stock solution was taken in 25 mL volumetric flask placed in the direct sunlight for 1 h. After completion of the stress the 25 mL flask was completed up to the mark with mobile phase.

### Robustness

Premeditate variations were performed in the experimental conditions of the proposed method to assess the method robustness. For this intention, minor changes were made in mobile phase composition, flow rate and pH of buffer solution. The effect of these changes on chromatographic parameters such as retention time, tailing factor and number of theoretical plates was then measured.

### Limit of detection and limit of quantitation

Limit of quantitation and limit of detection values were determined by the signal-to-noise (S/N) approach. Limit of quantitation is the concentration of the analyte that give a signal-to-noise (S/N) ratio of 10:1 at which analyte can be readily quantified with accuracy and precision. Limit of detection is the concentration of the analyte that give signal-to-noise (S/N) ratio of 3:1 at which analyte can be readily detected. To investigate the limit of quantitation and limit of detection solutions of different concentrations were prepared by spiking know amounts of moxifloxacin and prednisolone into excipients (benzalkonium chloride and sodium chloride). Each solution was prepared according to the procedure and analysed repeatedly to determine the S/N ratio. The average S/N ratio from all the analyses at each concentration level was used to calculate the limit of quantitation and limit of detection. The concentration level that gives an S/N ratio of about 10:1 at which analytes can be readily quantified with accuracy and precision was reported as the limit of quantitation. The concentration level that gives an S/N ratio of about 3:1 at which analytes can be readily detected was reported as the limit of detection.

## Results and discussion

In reverse phase liquid chromatographic separation of pharmaceutical drugs, choice of a stationary phase depends on the chemical structures of the target analytes. Moxifloxacin and prednisolone drugs have high carbon to heteroatom ratio and therefore can be separated through C8 or C18 stationary phase based mainly on their overall hydrophobicity. Due to the presence of π electrons, moxifloxacin and prednisolone also considered to be separated using phenyl stationary phase involving π- π interactions between the phenyl groups in the stationary phase and any unsaturated bonds in moxifloxacin and prednisolone structures. Both the drugs also contains polar functional groups (−COOH or ^–^OH etc.) and may be separated using cyano stationary phase involving dipole-dipole interactions.

In this work we proposed a simple, fast, and accurate stability indicating RP-HPLC method for simultaneous determination of moxifloxacin and prednisolone. For optimization of the chromatographic conditions and to obtain symmetrical peaks with better resolution and with no peak impurity, various chromatographic conditions such as composition of mobile phase, mobile phases with different pH, stationary phases with different packing materials (Hypersil BDS C8, Hypersil ODS C18, Hypersil BDS Phenyl-2, and Hypersil BDS Cyano) and configurations (10, 15, 25 cm columns) were applied to moxifloxacin and prednisolone combination.

### Optimization of mobile phase, stationary phase and pH

Method development process was initiated with different ratios (20:80, 30:70, 40:60 and 50:50) of water and methanol at different pH. With methanol and water broad peaks of prednisolone and asymmetrical peaks of moxifloxacin were obtained on all four different stationary phases (Hypersil BDS C8, Hypersil ODS C18, Hypersil BDS Phenyl-2, and Hypersil BDS Cyano) with long retention of prednisolone on C18 column. Increase in the temperature of column oven to 50°C did not improve peak tailing and broadening of analytes. The peak tailing of moxifloxacin may be due to the chelate formation of metal ion impurity of the stationary phases with the carboxyl group of C-3 and oxygen atom of C-4 of moxifloxacin or it may be caused due to unwanted interactions between nitrogen atoms (of moxifloxacin) and silanol residues of stationary phases. The peak broadening of prednisolone may be due to low polarity of the mobile phase. Then polarity of the mobile phase was increased by using phosphate buffer in the mobile phase composition. Further chromatographic experiments were performed on four different stationary phases (Hypersil BDS C8, Hypersil ODS C18, Hypersil BDS Phenyl-2, and Hypersil BDS Cyano) using methanol: phosphate buffer as mobile phase along with (0.1%v/v) triethylamine (as silanol blocker). Highly symmetrical and sharp peaks of moxifloxacin and prednisolone were obtained with methanol: 0.018 M phosphate buffer containing 0.1%v/v triethylamine (62:38,v/v) on Hypersil BDS C8 columns (with better resolution, capacity factor, peak shapes and theoretical plates) as compared to other stationary phases (Hypersil ODS C18, Hypersil BDS Phenyl-2, and Hypersil BDS Cyano). The variations in the composition of the mobile phase and dissimilar stationary phases had substantial influences on peak shape, tailing factor, retention factor, theoretical plates and resolution.

**Figure 2:**
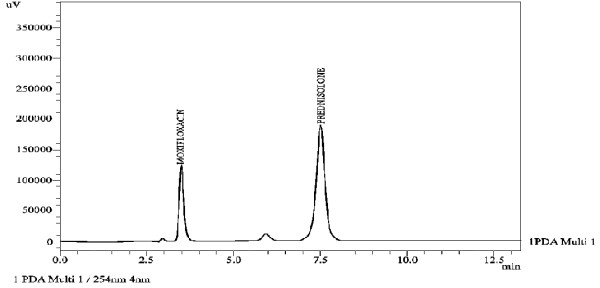
Chromatogram of moxifloxacin and prednisolone in pharmaceutical formulations.

In order to optimize the appropriate pH of the phosphate buffer solution, chromatographic experiments were performed at four different pH (2.8, 3.5, 4.5 and 6.5) values of the buffer solution. Mobile phase was prepared in the ratio of methanol: phosphate buffer (62: 38, v/v) containing 0.1%v/v triethylamine. Hypersil BDS C8 column was used as stationary phase to optimize the appropriate pH. Highly symmetrical and sharp peaks of moxifloxacin and prednisolone were obtained at pH 2.8 and 3.5 by using methanol: 0.018 M phosphate buffer with better resolution, capacity factor, and theoretical plates at pH 2.8 as compared to pH 3.5 [Table 1]. Finally, methanol: phosphate buffer 0.018 M, pH 2.8 (62:38,v/v) was selected which provided symmetrical peaks with acceptable peak purity index of moxifloxacin and prednisolone using Hypersil BDS C8 column. Under the mentioned chromatographic conditions highly symmetrical and sharp peaks of moxifloxacin and prednisolone were obtained at retention times of 3.449, and 7.447 min, respectively [Figure 2].

**Table 1:** pH Optimization of phosphate buffer

**Mobile phase**	**Retention Capacity (k**^**/**^**)**	**Theoretical plates (N)**	**Tailing Factor (T)**	**Resolution (R)**	**Peak shape**
**Methanol: Phosphate Buffer**					
**pH 2.8 (62:38)**					
Moxifloxacin		4521	1.17		+++
Prednisolone	0.92	6568	1.01	13.66	+++
**Methanol: Phosphate Buffer**					
**pH 3.5 (62:38)**					
Moxifloxacin		4251	1.29		+++
Prednisolone	0.84	5784	1.18	12.24	+++
**Methanol: Phosphate Buffer**					
**pH 4.5 (62:38)**					
Moxifloxacin		4122	1.47		+++
Prednisolone	0.54	5541	1.14	9.45	---
**Methanol: Phosphate Buffer**					
**pH 6.5 (62:38)**					
Moxifloxacin		3801	1.40		+++
Prednisolone	0.41	4787	1.28	11.87	---

Chromatographic conditions, mobile phase methanol: 0.018 M phosphate buffer 62:38, pH 2.8, or 3.5, or 4.5, or 6.5, Column BDS Hypersil C8 (250 X 4.6, 5 μm), flow rate 1.5 mL min^-1^,

injection volume 20 μL, wavelength 254 nm, +++ is peak acceptable and --- is peak not acceptable.

The developed chromatographic method was validated using ICH guidelines [24]. Validation parameters included linearity, accuracy, precision, robustness, specificity, limit of detection and quantitation.

Linear calibration plots for the proposed method were obtained in concentration ranges of 20–80 μg mL^-1^ (20, 30, 40, 50, 60, 70 and 80 μg mL^-1^) for moxifloxacin and 40–160 μg mL^-1^ prednisolone (40, 60, 80, 100, 120, 140 and 160 μg mL^-1^). The linear regression equation (r^2^ ≥ 0.998) for moxifloxacin was found to be Y = 22711 X + 23429 (Y = aX + b) in which Y is the dependent variable, X is independent variable, 22711 is slope (denoted by a) which shows change in dependent (Y) variable per unit change in independent (X) variable and 23429 (denoted by b) is the Y-intercept i.e., the value of Y variable when X = 0. The linear regression equation (r^2^ ≥ 0.999) for prednisolone was Y = 69932 X + 73339 (Y = aX + b) in which 69932 is the slope (a) and 73339 is the Y-intercept (a point of the Y-coordinate where a given line intersects the Y-axis) at which X is equal to zero. It defines the elevation of the line. The Y-intercept provides with an estimate of the variability of the method. For example, the ratio percent of the Y-intercept with the variable data at nominal concentration is used to estimate the method variability.

The limit of detection (LOD) and quantitation (LOQ) were determined by making serials of dilutions. LOD was found to be 0.088 μg mL^-1^ and 0.175 μg mL^-1^ for moxifloxacin and prednisolone, respectively (signal to noise ratio of 3:1). LOQ was found to be 0.284 μg mL^-1^ and 0.559 μg mL^-1^ for moxifloxacin and prednisolone, respectively (signal to noise ratio of 10:1).

Accuracy of the developed method was performed by the standard addition and synthetic mixture techniques. Three levels of solutions (50, 100 and 150%) of the nominal analytical concentrations were prepared and analysed by the developed method. Percentage recoveries along with standard deviation and relative standard deviations for each analyte are given in [Table 2]. Recovery studies showed the method to be highly accurate and suitable for intended use.

**Table 2:** Accuracy of the proposed HPLC method

**Drugs**	**Spiked concentration(μg mL**^**-1**^**)**	**Standard addition**	**Synthetic mixtures**
		**Measured concentration(μg mL**^**-1**^**) ± SD; RSD (%)**	**Measured concentration(μg mL**^**-1**^**) ± SD; RSD (%)**
Moxifloxacin	25.0	25.2 ± 0.1; 0.1	24.8 ± 0.2; 0.3
	50.0	50.4 ± 0.9; 1.4	50.6 ± 0.4; 0.6
	75.0	76.8 ± 1.1; 1.7	74.8 ± 1.1; 1.2
Prednisolone	50.0	50.9 ± 0.7; 0.7	50.1 ± 0.9; 0.1
	100.0	98.4 ± 0.9; 0.7	99.7 ± 0.8; 1.3
	150.0	147.9 ± 0.7; 0.2	150.0 ± 0.5; 0.7

*n* = Average of 5 analysis, Chromatographic conditions: mobile phase methanol: 18 mM phosphate buffer 62:38, v/v, pH 2.8, Column BDS Hypersil C8 (250 X 4.6, 5 μm), flow rate 1.5 mL min^-1^, injection volume 20 μL, wavelength 254 nm.

Intra-day precision was determined by injecting five standard solutions of three different concentrations on the same day and inter-day precision was determined by injecting the same solutions for three consecutive days. Relative standard deviation (RSD %) of the peak area calculated to represent precision. The results of intra-day and inter-day precision are presented in [Table 3].

**Table 3:** Intra-Day and Inter-Day precision of the proposed HPLC method

**Drugs**	**Actual concentration(μg mL**^**-1**^**)**	**Intra-day precision**	**Inter-day precision**
		**Measured concentrations; RSD (%)**	**Measured concentrations; RSD (%)**
Moxifloxacin	25.0	24.8 ± 0.6; 1.7	25.3 ± 0.4; 1.1
	50.0	50.5 ± 0.7; 0.2	51.2 ± 0.8; 1.0
	75.0	74.1 ± 0.4; 1.5	75.7 ± 1.2; 1.5
Prednisolone	50.0	50.0 ± 0.1; 0.3	50.4 ± 1.7; 1.0
	100.0	101.5 ± 0.4; 0.9	102.0 ± 0.9; 0.5
	150.0	153.1 ± 0.8; 1.7	151.8 ± 0.7; 1.6

*n* = Average of 5 analysis, Chromatographic conditions: mobile phase methanol: 18 mM phosphate buffer 62:38, v/v, pH 2.8, Column BDS Hypersil C8 (250 X 4.6, 5 μm), flow rate 1.5 mL min^-1^, injection volume 20 μL, wavelength 254 nm.

Robustness of the method was performed by slightly varying the chromatographic conditions. The results showed that slight variations in chromatographic conditions had negligible effect on the chromatographic parameters [Table 4 and Table 5].

**Table 4:** Robustness study of moxifloxacin

**Chromatographic conditions**	**Assay %**	**t**_**R**_**(min)**	**Theoretical plates**	**Tailing**
Methanol:buffer (64:36)	99.1	3.241	4347	1.26
Methanol:buffer (62:38)	102.0	3.446	4328	1.26
Methanol:buffer (60:40)	100.5	3.615	4498	1.24
Flow rate (1.3 mL/min)	100.8	3.825	4448	1.26
Flow rate (1.5 mL/min)	98.4	3.447	4319	1.26
Flow rate (1.7 mL/min)	98.2	3.089	4358	1.26
Buffer (pH 2.6)	101.0	3.442	4348	1.24
Buffer (pH 2.8)	100.9	3.446	4442	1.26
Buffer (pH 3.0)	101.2	3.443	4488	1.24

**Table 5:** Robustness Study of Prednisolone

**Chromatographic Conditions**	**Assay %**	**t**_**R**_**(min)**	**Theoretical plates**	**Tailing**
Methanol:buffer (64:36)	99.2	6.947	6328	1.01
Methanol:buffer (62:38)	100.8	7.447	6277	1.01
Methanol:buffer (60:40)	99.7	8.499	6341	1.01
Flow rate (1.3 mL/min)	99.4	8.433	6358	1.01
Flow rate (1.5 mL/min)	100.5	7.447	6249	1.02
Flow rate (1.7 mL/min)	100.4	6.569	6371	1.01
Buffer (pH 2.6)	100.4	7.497	6395	1.00
Buffer (pH 2.8)	99.9	7.447	6354	1.02
Buffer (pH 3.0)	98.8	7.498	6248	1.00

**Figure 3:**
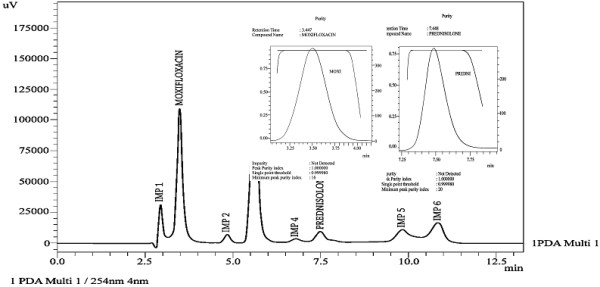
Chromatogram of moxifloxacin and prednisolone under acidic stress.

**Figure 4:**
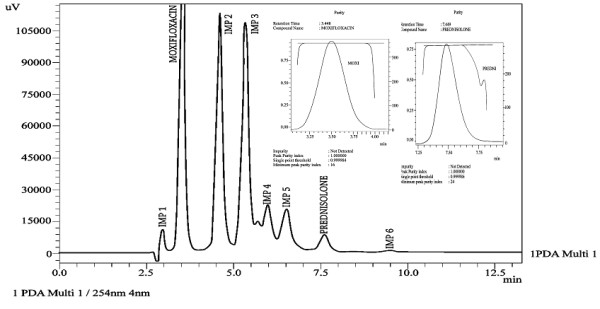
Chromatogram of moxifloxacin and prednisolone under basic stress.

**Figure 5:**
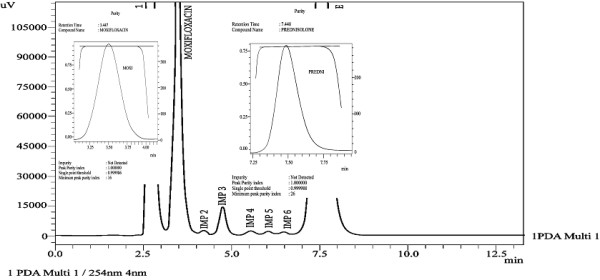
Chromatogram of moxifloxacin and prednisolone under oxidative stress.

**Figure 6:**
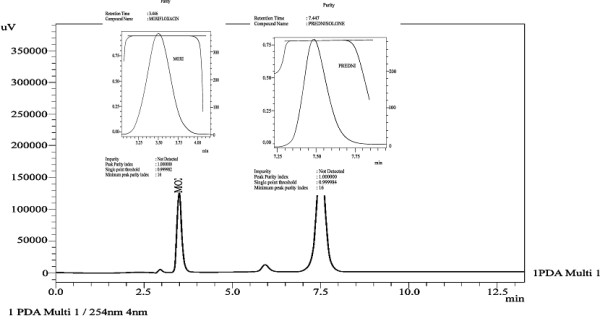
Chromatogram of moxifloxacin and prednisolone under thermal stress.

**Figure 7:**
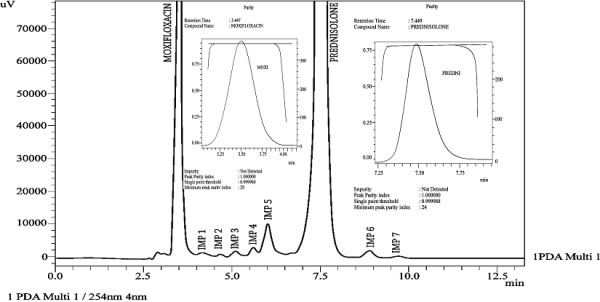
Chromatogram of moxifloxacin and prednisolone under photolytic stress.

Specificity of the developed method was evaluated by applying different stress conditions (acid, base, oxidation, thermal and photolytic) to moxifloxacin and prednisolone in combination form. The chromatograms under different stress conditions are showed in [Figures 3, 4, 5, 6, 7]. The results of stress studies are given in [Table 6].

**Table 6:** Stress Testing Results of Moxifloxacin and Prednisolone

**Nature of stress**	**Storage conditions**	**Time (h)**	**Amount of moxifloxacin**	**Amount of prednisolone**	**Extent of decomposition**
			**Remaining ± RSD (%)**	**Remaining ± RSD (%)**	
5 M HCl	(40°C/75% RH)	16	86.3 ± 3.4 **(PPI = 1.0000)**	4.8 ± 3.0 **(PPI = 1.0000)**	**Substantial**
5 M NaOH	(22°C/58% RH)	0.67	100.0 ± 2.1 **(PPI = 1.0000)**	3.4 ± 2.7 **(PPI = 1.0000)**	**Substantial**
6% H_2_O_2_	(40°C/75% RH)	16	96.4 ± 1.5 **(PPI = 1.0000)**	88.3 ± 1.4 **(PPI = 1.0000)**	**Substantial**
Thermal	(40°C/75% RH)	144	101.3 ± 1.4 **(PPI = 1.0000)**	100.5 ± 1.9 **(PPI = 1.0000)**	**None**
	(40°C/75% RH)	288	97.5 ± 1.1 **(PPI = 1.0000)**	97.2 ± 1.5 **(PPI = 1.0000)**	**None**
Dry Heat	(105°C)	7	93.7 ± 1.7 **(PPI = 1.0000)**	84.1 ± 1.4 **(PPI = 1.0000)**	**Substantial**
Photolytic	Sunlight	1	88.5 ± 1.9 **(PPI = 1.0000)**	94.5 ± 2.5 **(PPI = 1.0000)**	**Substantial**

*n* = Average of 3 determinations, *PPI* = Peak Purity Index, Chromatographic conditions: mobile phase Methanol: 18 mM phosphate buffer 62:38, v/v, pH 2.8, Column BDS Hypersil C8 (250 X 4.6, 5 μm), flow rate 1.5 mL min^-1^, injection volume 20 μL, wavelength 254 nm.

All the stress conditions applied were enough to degrade both the drugs. Comparison of the two drugs showed that moxifloxacin is more stable as compared to prednisolone. Under acidic conditions prednisolone was degraded up to 95.2% and moxifloxacin was degraded up to 13.7%. Under basic stress prednisolone was degraded up to 96.6% and moxifloxacin was found to be stable under basic stress. Under oxidative stress prednisolone was degraded up to 11.7% and moxifloxacin was found to be stable under oxidative stress. Under thermal stress (dry heat) moxifloxacin was degraded up to 6.3% and prednisolone was degraded up to 15.9%. Under photolytic stress prednisolone and moxifloxacin were degraded up to 5.5% and 11.5%, respectively. From these stress studies it is thus concluded that prednisolone and moxifloxacin drugs are not stable in basic, acidic, oxidative, thermal and photolytic stress conditions.

In addition to the percentage degradation of each drug, a number of degradation products (impurities) were produced under acidic (6 impurity/degradation peaks with IMP 3 as major degradation peak), basic (6 impurity/degradation peaks with IMP 2 and IMP 3 as major degradation peaks), oxidative (6 impurity/degradation peaks with IMP 1 as degradation peak) and photolytic stress (7 impurity/degradation peaks with IMP 5 as major degradation peak) conditions. The developed method effectively separated the degradation products or impurities (6 impurity peaks under acidic stress, 6 impurity peaks under basic stress, 6 impurity peaks under oxidative stress, 7 impurity peaks under photolytic stress,) from analyte peaks [Figures 3, 4, 5, 6, 7]. Therefore, the developed method is to be considered highly specific for intended use. Application of the developed method was checked by analyzing the moxifloxacin and prednisolone in commercially available pharmaceutical products. The results are provided in [Table 7] which showed high percentage recoveries and low RSD (%) values for both analytes.

**Table 7:** Assay results of moxifloxacin and prednisolone in commercial eye drops

**Products**	**Ingredient**	**Label value (mg per mL)**	**% Recovery ±RSD (%)**
**Eye drops**			
Occumox P	Moxifloxacin	5	100.3 ± 0.3
	Prednisolone	10	99.0 ± 0.7
Moftrex P	Moxifloxacin	5	100.7 ± 0.1
	Prednisolone	10	98.9 ±0.7

*n* = Average of 10 determinations, Chromatographic conditions: mobile phase Methanol: 18 mM phosphate buffer 62:38, v/v, pH 2.8, Column BDS Hypersil C8 (250 X 4.6, 5 μm), flow rate 1.5 mL min^-1^, injection volume 20 μL, wavelength 254 nm.

## Conclusion

A simple, fast and accurate stability indicating RP-HPLC method is described for simultaneous determination of moxifloxacin and prednisolone in pharmaceutical formulations. The developed method was validated by testing its linearity, accuracy, precision, limits of detection and quantitation and specificity. The method is simple, fast and is without the use of ion pair or any derivatization reagent. The method is good enough to separate the peaks of active pharmaceutical ingredients (APIs) from the degradation products (produced during forced degradation studies). So, it is concluded that the method can be successfully used for any kind of stability and validation studies.

## Competing interests

The authors declare that they have no competing interests.

## Authors’ contributions

SNR: Participated in method development and optimization, collect the literature review, performed forced degradation studies and wrote the manuscript. IUK: proposed, planned and supervised the whole work. SSR and IM: Participated in the method validation. All authors read and approved the final manuscript.
